# Kidney Stone Risk in Normocalcemic Hyperparathyroidism before and after Parathyroid Surgery

**DOI:** 10.1155/2024/1252724

**Published:** 2024-07-17

**Authors:** Jie Tang, Kamil Malshy, Gyan Pareek

**Affiliations:** ^1^ Division of Kidney Diseases and Hypertension Alpert Medical School of Brown University, Providence, RI, USA; ^2^ Division of Urology Alpert Medical School of Brown University, Providence, RI, USA

## Abstract

The higher risk for kidney stone in patients with primary hyperparathyroidism is well-documented; stone risk in patients with normocalcemic primary hyperparathyroidism (NPHPT) remains unclear. We present a case of recurrent calcium kidney stones in a patient with severe idiopathic hypercalciuria and NPHPT. The surgical resection of the parathyroid adenoma failed to reduce kidney stone risk (based on the 24-hr urine study) and kidney stone burden (based on ultrasound). This unique case examines the impact of surgical resection of an ectopic parathyroid adenoma on stone risk in a patient with NPHPT and recurrent calcium kidney stones.

## 1. Introduction

Parathyroid hormone (PTH) plays an essential role in calcium and phosphorus homeostasis. High levels of PTH in primary hyperparathyroidism (PHPT) significantly increase the risk for calcium kidney stone formation, preceded by hypercalcemia and hypercalciuria [1, 2]. Normocalcemic hyperparathyroidism (NPHPT) is considered a variant of primary hyperparathyroidism characterized by normal serum calcium levels [3]. Unlike PHPT, the effect of NPHPT on kidney stone risk has not yet been elucidated. In a study of 414 kidney stone formers with normal kidney function, 9.6% had NPHPT, but there was no distinct pattern of abnormalities in stone risk profile associated with NPHPT [4]. However, the study was cross-sectional with many potential confounders. Thus far, no studies have examined the changes in stone risk after definitive surgical intervention. Here, we present a patient with severe hypercalciuria and recurrent calcium kidney stones, who was diagnosed with NPHPT associated with an ectopic adenoma. We compared her stone risk profile and burden before and after surgical resection of the adenoma.

## 2. Case Presentation

**Figure 1:**
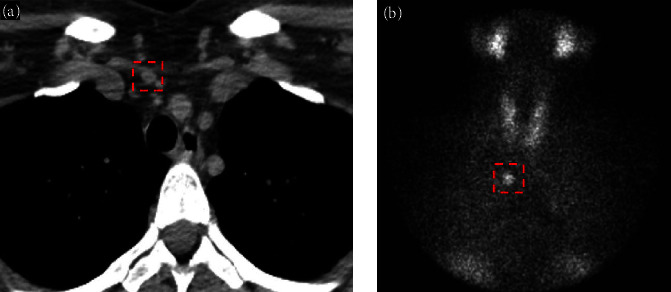
Transversal view of the CT scan (a) highlighting the PTH secreting adenoma at the right upper chest level, along with (b) parathyroid scintigraphy showed a focus of increased 99mTc-sestamibi uptake corresponding to an ectopic parathyroid functioning adenoma.

**Figure 2:**
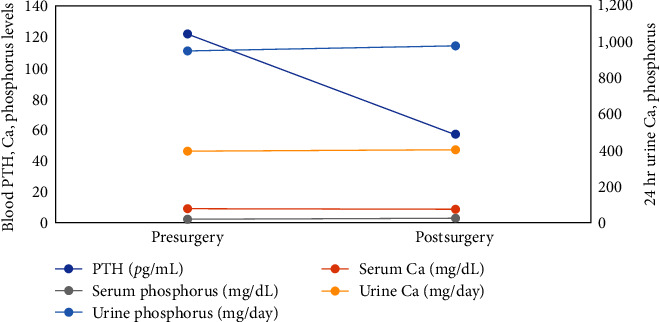
Changes in blood and urine calcium and phosphorus concentrations 7 months before and 8 months after parathyroid adenoma resection.

A 43-year-old Caucasian female presented with recurrent calcium kidney stones. Her kidney stone history started in her late 20s and her last colicky kidney stone attack was 3 months prior. Computerized tomography (CT) scan at the time showed mild right hydroureteronephrosis extending to the right distal ureter with a 4 mm stone at the ureterovesical junction. Several additional intrarenal stones were also observed bilaterally. She was treated conservatively and passed her 4 mm stone in a couple of days without surgical intervention. She was suspected of having calcium-based kidney stone due to high supersaturation of calcium oxalate in her previous urine collection, although no stones were ever collected for direct analysis. She has no other significant past medical history and was not taking any medications or dietary supplements. Her family history was strongly positive for kidney stone with the disease affecting both her parents and siblings. She is overweight with a BMI of 28.7 kg/m^2^. The physical exam was otherwise unremarkable. Laboratory workup revealed an elevated plasma intact PTH (iPTH) concentration at 138 pg/mL (Table 1). Her serum calcium concentration was 9.6 mg/dL with a serum albumin concentration of 4.2 g/dL. Her ionized calcium concentration was 5.1 mg/dL. Retrospectively, her serum calcium levels have always been normal over the past 5 years. Her serum creatinine (Cr) concentration was 0.6 mg/dL, blood total CO_2_ was 24 meq/L. Blood concentrations of other electrolytes (including magnesium and phosphorus) and uric acid were all normal. Her blood 25-hydroxy-vitamin D (25(OH)D) level was 20 ng/mL with a normal 1,25-dihydroxy-vitamin D (1,25(OH)_2_D) concentration (Table 1). Twenty-four hour urine kidney stone risk panel performed 4 months later showed severe hypercalciuria (447 mg) with normal urine sodium, potassium, and citrate. Her point-of-care kidney ultrasound showed a single 4 mm left intrarenal stone and normal-appearing kidneys without evidence of hydronephrosis or nephrocalcinosis. She started on cholecalciferol 3,000 international units daily and was instructed to optimize dietary calcium intake. Seven months after her initial visit, she presented again with left flank pain. Repeat plasma iPTH remained elevated at 123 pg/mL, despite a significantly improved 25(OH)D level. Other laboratory values were similar to her initial visit (Table 1). A repeat kidney ultrasound showed new mild left hydronephrosis with another 7 mm stone in her left kidney. She was again treated conservatively and was able to pass the stone without surgical intervention. Due to her severe hypercalciuria, persistent elevation of PTH, and frequent colicky stone attacks a Tc-99m Sestamibi parathyroid scan was performed which demonstrated a 9 × 6 × 12-mm ectopic parathyroid adenoma within the superior mediastinum (Figure 1). She subsequently underwent surgical parathyroid adenoma resection 10-month after her initial presentation, prompted by the triad of rapidly progressing kidney stone disease, elevated PTH levels, and the presence of an ectopic adenoma. The aim was to address the origin of hyperparathyroidism, despite normocalcemia. She was seen 1 month after the surgery with plasma iPTH normalized to 71 pg/mL. Repeat 24-hr urine kidney stone panel performed 8 and 27 months after the surgery failed to show any improvement in stone risk profile (Table 1). To further investigate her persistent hypercalciuria after surgery, we conducted split urine Ca/Cr test (random overnight fasting and postprandial). While the fasting ratio showed a normal result of 0.07 (urine Ca = 13.6 mg/dL; urine Cr = 192 mg/dL), the postprandial test demonstrated an elevated ratio of 0.24 (urine Ca = 25.4 mg/dL; urine Cr = 110 mg/dL). Next, a semifasting 24-hr urine collection was performed, which yielded a urine Ca of 220 mg/day. Both tests ruled out fasting hypercalciuria. Radiographically, kidney ultrasound again showed worsening stone burden with a new 4 mm stone in her right kidney in addition to her 7 mm left intrarenal stone which was unchanged. Three months later, she had another colicky attack.  Figure 2 also illustrates the changes in blood and urine calcium and phosphorus before and after the parathyroid adenoma resection.

## 3. Discussion

NPHPT is a recognized variant of PHPT characterized by normal serum calcium concentration. Our patient met these diagnostic criteria with persistently normal serum calcium concentrations and the presence of an ectopic parathyroid adenoma. Her vitamin D had been adequate, with serum 25(OH)D over 20 ng/mL and normal serum 1,25(OH)2D. She had normal kidney function and no evidence of gastrointestinal disorders causing calcium malabsorption. She is not taking any medication that can stimulate parathyroid release and has normal serum magnesium and phosphorus. Of note, our patient did have persistent hypercalciuria, which is considered by some as an exclusion criteria for the diagnosis of NPHPT. This is due to the concern of secondary parathyroid stimulation as a result of negative calcium balance, i.e., in cases of primary renal calcium wasting or bone loss. However, in this patient, the evidence supporting a diagnosis of primary hyperparathyroidism is overwhelming. First, an ectopic PTH-secreting adenoma was identified, followed by persistently normal PTH levels for over 2 years after surgical resection, despite the presence of hypercalciuria. In secondary, hyperparathyroidism due to renal leak syndrome, for instance, surgical treatment would not be able to maintain normal PTH levels over time. Second, the normalized urine calcium excretion under fasting argues against a primary calcium-wasting state. To our knowledge, this is the first reported case of a patient with NPHPT and recurrent calcium kidney stones, who underwent parathyroid adenoma resection. The surgical resection of parathyroid adenoma failed to improve kidney stone risk profile based on the 24-hr urine study and kidney stone burden based on kidney ultrasound.

Although NPHPT was first described over a decade ago [5], its pathogenesis remains poorly defined. Some consider it as an early and milder form of PHPT given observations that a portion of such patients developed hypercalcemia over time [3, 6]. Others consider it a unique variant with partial tissue resistance to PTH [7]. Its association with the risk of kidney stone disease is far from being established. Despite the fact that kidney stone disease has been well represented as a condition associated with a diagnosis of NPHPT [7, 8, 9, 10, 11], all the studies referenced were cross-sectional, lacked in-depth investigation of blood and urine stone risk markers, and assessment of actual stone burden. A referral or selection bias was also highly suspected as plasma iPTH was routinely measured in patients with calcium kidney stone disease. As a matter of fact, in a small cohort of nonstone formers with NPHPT, plasma iPTH levels did not correlate with urinary calcium concentration [6]. In a study of 414 prevalent kidney stone formers, 9.6% had NPHPT, all had detailed clinical history along with relevant blood tests and 24-hr urine analyses. Investigators failed to identify any specific patterns of stone risk profiles associated with NPHPT, all except one had normal urine calcium excretion [4]. Therefore, although a significant proportion of patients diagnosed with NPHPT had a history of kidney stone disease, the causal relationship between the two conditions remains to be established.

Our patient had severe underlying hypercalciuria which prompted a workup for hyperparathyroidism. In patients with PHPT, hypercalciuria is a main renal manifestation and parathyroidectomy will reduce urinary calcium excretion [12, 13]. However, in NPHPT, the occurrence of hypercalciuria is rare [4]. Although theoretically, it could be a direct result of excessive PTH as in cases of PHPT, the presence of nonfasting hypercalciuria in our case could also be a coincidental finding, like other cases of enteric hypercalciuria without PTH derangements. Renal phosphorus excretion is also worth mentioning, especially in cases suspected of a correlation with high PTH. PTH decreases phosphate reabsorption at the proximal convoluted tubule leading to phosphorus wasting [14]. Therefore, inappropriately low serum phosphorus concentration is common in patients with PHPT [15]. However, unexpectedly, curative parathyroidectomy did not change urinary phosphorus excretion in a study of seven stone formers with PHPT [12]. The effect of excess PTH on urine phosphorus in NPHPT has not been reported. In our case, no change in urinary phosphorus excretion was found after curative parathyroid adenoma resection.

Surgical management of NPHPT appears to improve bone-related outcomes, i.e., higher bone mineral density [16, 17], but its impact on the actual kidney stone burden remains unknown. Here, through this case, we show that the trajectory of change in kidney stone burden did not alter after adenoma resection and normalization of plasma iPTH. This is in discordance with a small pilot study examining the effect of cinacalcet on kidney stone burden in six kidney stone formers with NPHPT [18]. In that study, cinacalcet at doses that normalized PTH levels reduced the number and diameter of kidney stones after 10 months. However, the study had serious limitations. In addition to the small number of participants, hidden confounders, the study adopted kidney ultrasound as an imaging modality to assess the stone burden which is prone to subjective error especially when the study was unblinded. A publication bias in this type of study is also highly suspected. Furthermore, it is also possible that the observed effect could be due to other actions of cinacalcet independent of PTH lowering. Calcium-sensing receptor is highly expressed in the kidney integrating signals from both basolateral interstitium and apical tubular fluid. Its activation can promote calcium delivery to the distal nephron, acidify urine, and down-regulate aquaporin-2 expression leading to polyuria, reducing kidney stone risk [19, 20]. In our case, the resection of parathyroid adenoma did not appear to have a major impact on kidney stone burden despite a complete normalization of plasma iPTH. Nevertheless, clinical experience with NPHPT among kidney stone formers is limited. Although parathyroidectomy might be undertaken to reduce kidney stone burden in such cases, existing evidence regarding the actual surgical benefit has been limited [17]. Further research on this topic is urgently needed.

In summary, we show through this case report, for the first time that curative surgical parathyroid ectopic adenoma resection did not change kidney stone risk profile in a stone former with NPHPT. The presence of baseline idiopathic hypercalciuria is likely contributing to the stone formation. Since so little is known about the actual relationship between kidney stone disease and NPHPT, more research is needed to assess the causal relationship between these two conditions.

## Figures and Tables

**Table 1:** Relevant laboratory findings before and after parathyroid adenoma resection.

Laboratory results	Preresection			Postresection	
	14 months prior	10 months prior	7 months prior	8 months after	27 months after
Plasma iPTH (pg/mL)	138	136	123	58	57
Serum					
Creatinine (mg/dL)	0.6	0.6	0.6	0.6	0.46
Calcium (mg/dL)	9.6	9.4	9.7	9.1	8.7
Magnesium (meq/L)	1.7	1.5	1.6	1.7	Not done
Phosphorus (mg/dL)	3.1	2.5	2.9	3.0	Not done
25(OH)D (ng/mL)	20	18	29	34	26.6
1,25(OH)_2_D (pg/mL)	76	62	59	61	Not done
Urine					
Calcium (mg/day, mg/kg/day ^*∗*^)	Not done	447, 5.54	401, 4.97	409, 5.07	393, 4.33
Phosphorus (mg/day)	Not done	877	958	992	554
Oxalate (mg/day)	Not done	29	21	24	40
Citrate (mg/day)	Not done	794	778	640	774
Sodium (mmol/day)	Not done	151	102	138	145
Potassium (mmol/day)	Not done	45	48	40	44
Creatinine (mg/day)	Not done	1,141	1,081	1,276	1,452

^*∗*^Adjusted to patients' weight.

## Data Availability

Records and data pertaining to this case report are stored electronically at the Division of Kidney Diseases and Hypertension, Alpert Medical School, Brown University in Providence, USA, and can be provided by the corresponding author upon reasonable request.

## References

[1] Silva B. C., Cusano N. E., Bilezikian J. P. (2018). Primary hyperparathyroidism. *Best Practice & Research Clinical Endocrinology & Metabolism*.

[2] Mollerup C. L., Vestergaard P., Frøkjaer V. G., Mosekilde L., Christiansen P., Blichert-Toft M. (2002). Risk of renal stone events in primary hyperparathyroidism before and after parathyroid surgery: controlled retrospective follow up study. *BMJ*.

[3] Schini M., Jacques R. M., Oakes E., Peel N. F. A., Walsh J. S., Eastell R. (2020). Normocalcemic hyperparathyroidism: study of its prevalence and natural history. *The Journal of Clinical Endocrinology and Metabolism*.

[4] Dimkovic N. B., Wallele A. A., Oreopoulos D. G. (2002). Renal stone disease, elevated iPTH level and normocalcemia. *International Urology and Nephrology*.

[5] Eastell R., Brandi M. L., Costa A. G., D’Amour P., Shoback D. M., Thakker R. V. (2014). Diagnosis of asymptomatic primary hyperparathyroidism: proceedings of the fourth international workshop. *The Journal of Clinical Endocrinology & Metabolism*.

[6] Silverberg S. J., Bilezikian J. P. (2003). “Incipient” primary hyperparathyroidism: a “forme fruste” of an old disease. *The Journal of Clinical Endocrinology & Metabolism*.

[7] Maruani G., Hertig A., Paillard M., Houillier P. (2003). Normocalcemic primary hyperparathyroidism: evidence for a generalized target-tissue resistance to parathyroid hormone. *The Journal of Clinical Endocrinology & Metabolism*.

[8] Lowe H., McMahon D. J., Rubin M. R., Bilezikian J. P., Silverberg S. J. (2007). Normocalcemic primary hyperparathyroidism: further characterization of a new clinical phenotype. *The Journal of Clinical Endocrinology & Metabolism*.

[9] Marques T. F., Vasconcelos R., Diniz E., Rêgo D., Griz L., Bandeira F. (2011). Normocalcemic primary hyperparathyroidism in clinical practice: an indolent condition or a silent threat?. *Arquivos Brasileiros de Endocrinologia & Metabologia*.

[10] Amaral L. M., Queiroz D. C., Marques T. F., Mendes M., Bandeira F. (2012). Normocalcemic versus hypercalcemic primary hyperparathyroidism: more stone than bone?. *Journal of Osteoporosis*.

[11] Tordjman K. M., Greenman Y., Osher E., Shenkerman G., Stern N. (2004). Characterization of normocalcemic primary hyperparathyroidism. *The American Journal of Medicine*.

[12] Pak C. Y. (1979). Effect of parathyroidectomy on crystallization of calcium salts in urine of patients with primary hyperparathyroidism. *Investigative Urology*.

[13] Walker M. D., Silverberg S. J. (2018). Primary hyperparathyroidism. *Nature Reviews Endocrinology*.

[14] Knox F. G., Haas J. A., Lechene C. P. (1976). Effect of parathyroid hormone on phosphate reabsorption in the presence of acetazolamide. *Kidney International*.

[15] Watson L. (1974). Primary hyperparathyroidism. *Clinics in Endocrinology and Metabolism*.

[16] Koumakis E., Souberbielle J.-C., Payet J. (2014). Individual site-specific bone mineral density gain in normocalcemic primary hyperparathyroidism. *Osteoporosis International*.

[17] Bilezikian J. P., Silverberg S. J., Bandeira F. (2022). Management of primary hyperparathyroidism. *Journal of Bone and Mineral Research*.

[18] Brardi S., Cevenini G., Verdacchi T., Romano G., Ponchietti R. (2015). Use of cinacalcet in nephrolithiasis associated with normocalcemic or hypercalcemic primary hyperparathyroidism: results of a prospective randomized pilot study. *Archivio Italiano di Urologia e Andrologia*.

[19] Renkema K. Y., Velic A., Dijkman H. B. (2009). The calcium-sensing receptor promotes urinary acidification to prevent nephrolithiasis. *Journal of The American Society of Nephrology*.

[20] Riccardi D., Brown E. M. (2010). Physiology and pathophysiology of the calcium-sensing receptor in the kidney. *American Journal of Physiology-Renal Physiology*.

